# Multiple Mutations Associated with Emergent Variants Can Be Detected as Low-Frequency Mutations in Early SARS-CoV-2 Pandemic Clinical Samples

**DOI:** 10.3390/v14122775

**Published:** 2022-12-13

**Authors:** Jeffrey Kimbrel, Joseph Moon, Aram Avila-Herrera, Jose Manuel Martí, James Thissen, Nisha Mulakken, Sarah H. Sandholtz, Tyshawn Ferrell, Chris Daum, Sara Hall, Brent Segelke, Kathryn T. Arrildt, Sharon Messenger, Debra A. Wadford, Crystal Jaing, Jonathan E. Allen, Monica K. Borucki

**Affiliations:** 1 Lawrence Livermore National Laboratory, Livermore, CA 94550, USA; 2 Department of Biochemistry, Emory University School of Medicine, Atlanta, GA 30322, USA; 3 Lawrence Berkeley National Laboratory, US Department of Energy Joint Genome Institute, Berkeley, CA 94720, USA; 4 Viral and Rickettsial Disease Laboratory, California Department of Public Health, Richmond, CA 94804, USA

**Keywords:** severe acute respiratory syndrome coronavirus-2 (SARS-CoV-2), variant, mutation, sequence, quasispecies, iSNV, LoFreq, emergence, evolution

## Abstract

Genetic analysis of intra-host viral populations provides unique insight into pre-emergent mutations that may contribute to the genotype of future variants. Clinical samples positive for SARS-CoV-2 collected in California during the first months of the pandemic were sequenced to define the dynamics of mutation emergence as the virus became established in the state. Deep sequencing of 90 nasopharyngeal samples showed that many mutations associated with the establishment of SARS-CoV-2 globally were present at varying frequencies in a majority of the samples, even those collected as the virus was first detected in the US. A subset of mutations that emerged months later in consensus sequences were detected as subconsensus members of intra-host populations. Spike mutations P681H, H655Y, and V1104L were detected prior to emergence in variant genotypes, mutations were detected at multiple positions within the furin cleavage site, and pre-emergent mutations were identified in the nucleocapsid and the envelope genes. Because many of the samples had a very high depth of coverage, a bioinformatics pipeline, “Mappgene”, was established that uses both iVar and LoFreq variant calling to enable identification of very low-frequency variants. This enabled detection of a spike protein deletion present in many samples at low frequency and associated with a variant of concern.

## 1. Introduction

The emergence, spread, and evolution of severe acute respiratory syndrome coronavirus-2 (SARS-CoV-2) has been chronicled by the scientific community with greater speed and depth than any other human pathogen due to the advent of widespread genomic sequencing. Additionally, numerous websites and dashboards enable emergence of mutations to be visualized and contextualized [1,2,3]. RNA viruses such as SARS-CoV-2 evolve as a mutant spectra due to the high mutation rate that characterizes the evolutionary dynamics of these viruses [4], and deep sequencing is required to capture the genetic diversity of intra-host viral populations that shape infection outcome and variant emergence [5,6]. However, the genomic sequences used to populate the databases that scientists and public health officials rely on to study the transmission and evolution of SARS-CoV-2 represent the consensus (dominant) sequence detected in a sample. The sequencing methods used to generate consensus sequence data, multiplexed RT-PCR amplification of the viral genome [7], may also generate important information about genotypes that are present in clinical samples at low frequency. Data on subconsensus mutations that comprise the mutant spectra provide a rich source of genetic information about the emergence of viral variants. In this study, Illumina sequence data were derived from 90 COVID-positive, early pandemic clinical samples were analyzed for the presence and persistence of subconsensus mutations that later emerged in consensus sequences as the pandemic progressed.

SARS-CoV-2, a member of the *Betacoronavirus* genus and the *Coronaviridae* family, has a single-stranded, positive-sense RNA genome of ~30 kb [8]. The 5′ two-thirds of the genome codes for 16 non-structural proteins (nsp) which are derived from two open reading frames (orf1A and orf1b) via protease digestion of two overlapping polyproteins (Supplementary Figure S1). The 3′ one-third of the genome codes for the structural proteins including the Spike (S) protein, the Envelope (E) protein, the Membrane (M) protein, and the Nucleocapsid (N) protein, as well as multiple accessory proteins (ORF3a, ORF3b, ORF6, ORF7a, ORF7b, ORF8b, ORF9b and ORF10). The S protein largely determines the host range, immune evasion and virulence, and is the most thoroughly studied [9,10]. Similar to other viruses with an RNA genome, the RNA-dependent RNA polymerase (RdRp) is low fidelity and introduces errors at a high rate during replication. The error rate of CoVs is lowered by greater than 10-fold in SARS-CoV-2 and other coronaviruses due to the presence of the nsp14 gene which encodes a protein with 3′-5′ exonuclease proof-reading activity [11,12]; however, the error rate is still high enough to generate a mutant swarm of variant genotypes during replication within a host.

The presence and fate of mutant SARS-CoV-2 genotypes in clinical samples from early in the pandemic were explored via deep sequencing of 90 human clinical samples obtained from California Department of Public Health (CADPH) collected between February and July 2020. A bioinformatic pipeline was assembled to enable rapid analysis of the large datasets generated from high depth coverage, deep sequencing of the genome. The intra-host single nucleotide variants (iSNVs) that were detected were compared to consensus sequence data present in public databases (GISAID and NCBI) to determine if deep sequencing analysis provided insight into the persistence and eventual emergence of viral variant genotypes.

## 2. Materials and Methods

**RNA extraction**: Clinical respiratory specimens were either nasopharyngeal or oropharyngeal swabs in a viral transport medium or a universal transport medium. Samples were extracted using either the manual Qiagen QIAamp DSP Viral RNA Mini Kit or the Qiagen EZ1 DSP Virus Kit using the QIAgen EZ1 Advanced XL instrument (Qiagen Sciences, MD, USA).

**RT-qPCR**: Respiratory specimens collected prior to 21 May 2020 were tested for SARS-CoV-2 using the US Food and Drug Administration (FDA) Emergency Use Authorized (EUA) CDC 2019-Novel Coronavirus (2019-nCoV) Real-Time RT-PCR Diagnostic Panel (https://www.fda.gov/media/134922/download, accessed on 1 October 2021), as previously described. Briefly, extracted samples were added to an amplification reaction mix per the EUA assay (4X TaqPath 1-step RT-PCR master mix (Thermo Fisher, Carlsbad, CA, USA), 1.5 µL primer (N1, N2, or RNase P), 12.5 µL nuclease-free water, and 1 µL RNA) and one-step RT-PCR was performed (25 °C for 2 min, reverse transcription at 50 °C for 15 min, 95 °C for 2 min, followed by 45 cycles PCR with denaturing at 95 °C for 3 s and extension at 55 °C for 30 s). A confirmed positive sample was defined as having both N1 and N2 targets resulting at a C_t_ value of 40 cycles or less. After 21 May 2020, samples were extracted using the KingFisher Flex (Thermo Fisher Scientific) instrument according to the manufacturer’s instructions. These later samples were tested using the FDA EUA approved the Taqpath™ Multiplex Real-time RT-PCR test, which includes nucleoprotein (N) gene, spike (S) gene, and ORF1ab gene targets. Confirmed positives were those samples with 2 or more viral gene targets having C_t_ values of 37 or less. Only samples with a C_t_ below 28 (estimated to have greater than 1000 viral genomes per reaction) were selected for sequencing analysis.

**RT-PCR**. Viral RNA was amplified and sequenced using a version of the ARTIC protocol, nCoV-2019 sequencing protocol (RAPID barcoding, 1200bp amplicon) V.3 (https://www.protocols.io/view/ncov-2019-sequencing-protocol-rapid-barcoding-1200-bh7hj9j6; accessed on 13 October 2020). As described in Freed et al. (2020) [13], cDNA from each sample was amplified in a tiled approach using 2 highly multiplexed reactions using 2 different primer pools that span the genome. ARTIC 1200 bp primer sets were obtained from IDT (Integrated DNA Technologies) as two premixed primer sets (IDT Midnight Panel). The RT reaction was performed using the SuperScript IV VILO Master Mix kit (Invitrogen) with the first step using a DNase removal enzyme. The cDNA was amplified in a 50 µL PCR reaction using 2.5 µL cDNA and 2X Master mix (NEB). The PCR cycling program was 98 °C for 30 s, then 35 cycles of 98 °C for 15 s, 65 °C for 5 min. A negative PCR control for each PCR set was prepared using PCR grade water as a template. A PCR product from one of these negative controls was sequenced as a negative control. The PCR products from each reaction were pooled then were purified using AMPureXP beads (Beckman Coulter, Brea, CA, USA).

A no template negative control (NTC) was processed along with the other samples using the same PCR and sequencing protocols, and analyzed using the Mappgene pipeline. Most regions from the NTC had 0–20 reads mapped; however, a 120 nt region with >100 K depth of coverage was detected at nucleotide positions (nt pos) 21551-21670. The 5′ end of this region corresponds approximately to a forward primer (#22L) and occurs near the beginning of the S gene. A distinct peak in mapped reads at this region was also seen in coverage data from several experimental samples although deep coverage preceding and after this spike in mapped reads made the pattern less pronounced. Based on these data, any variant calls within this region were excluded from analysis. 

**Sequencing.** A total of 1 ng of the purified PCR product was tagmented using Nextera XT kit (Illumina, San Diego, CA, USA) and added unique dual sequencing indices by 10 cycles of PCR to make an Illumina sequencing library. The sequencing libraries were then purified and size selected for 300–500 bp with double SPRI using TotalPure NGS beads (Omega Bio-tek, Norcross, GA, USA). The prepared libraries were then quantified using KAPA Illumina library quantification kit (Roche, Budapest, Hungary) and run on a LightCycler 480 real-time PCR instrument (Roche). The quantified libraries were multiplexed and the pool of libraries was prepared for sequencing on the Illumina NovaSeq 6000 sequencing platform using NovaSeq XP v1.5 reagent kits (Illumina), S4 flow cell, and following a 2x150 indexed run recipe. 

**Bioinformatic analysis.** We developed Mappgene, (https://github.com/LLNL/mappgene v1.1.1, accessed 26 January 2022) a modular bioinformatics pipeline for high performance computing (HPC) (Supplementary Figure S2). It can convert high-throughput sequencing reads (FASTQ) into annotated variant calls in multiple formats (VCF/snpSIFT/bedGraph). Mappgene parses paired and unpaired FASTQ files and organizes them into directories for each subject. Then, it parallelizes processing tasks using the Parsl framework [14], enabling portability to almost any HPC platform. Software dependencies are stored in a Singularity container [15], ensuring software and version consistency. As a result, Mappgene can run at scales from a personal computer to hundreds of nodes on a supercomputer. To ease development, individual commands can be swapped in and out via Python scripting. Mappgene employs BWA-MEM [16] for read alignment, iVar for read trimming/filtering/variant calling, and LoFreq to call variants. 

**Base quality score recalibration.** Mappgene takes a conservative approach to recalibrating RTA3 Illumina read qualities. Initially, these read quality scores are labels for low, medium, and high-quality base bins as determined by Illumina. However, they are still represented as numeric Phred scores that sequence analysis tools interpret as actual error probabilities. This Phred score is meant to correspond to the average score for bases in low, medium, or high-quality base bins as determined by Illumina. Left as is, this could lead to both over- and underestimates of errors used to judge alignments, variant calls, and various filtering steps. Mappgene adjusts the Phred score to align with the expected lower boundary of the scores in the medium and high-quality bins (37 becomes 30 and 25 becomes 20).

**Read trimming and filtering**. iVar trim soft clips primer sequences from reads (soft clipping means the trimmed sequence is still in the bam file, but it is flagged as clipped and typically not included in length calculations). iVar trim trims bases from the 5′ end of reads if the average base quality drops below 20, using a 4 bp sliding window to calculate average base quality. iVar trim removes trimmed reads shorter than 31 bp and removes reads with mismatches in a primer region.

**Variant Calling**. iVar variants skips bases with quality scores less than 20. The minimum variant frequency iVar variants will detect is 1%. The LoFreq variant caller calls and filters variant calls according to its defaults which include a strand bias filter and minimum read count per locus of 10. iVar was used for variant calling for iSNV at 1% or greater and LoFreq was used for detection of variants <1%.

**Variant Summary.** A new software package MappgeneSummary (https://github.com/LLNL/MappgeneSummary; v0.6.3, accessed 26 January 2022) was developed to summarize the number and type of variants in the deeply sequenced samples. The bioinformatics pipeline used to analyze the data included 2 distinct variant callers, iVar and LoFreq. LoFreq uses a generalized binomial coverage aware sequence error model to give a statistically rigorous filter for variants occurring at less than 3% due to sequencing error. The program gives an orthogonal assessment to iVar, taking into consideration additional elements of sequencing quality such as variable depth of sequencing coverage not considered by iVar.

## 3. Results

### 3.1. Genome Sequencing and iSNV Detection Pipeline

Viral RNA was extracted from respiratory swab samples and the viral genome was amplified using a modified version ARTIC RT-PCR protocol [13], a well-established and widely used protocol that enables rapid enrichment of the viral genome. SARS-CoV-2 viral RNA was also quantified in each sample using Taqman PCR and the average C_t_ was 18.5.

**Figure 1:**
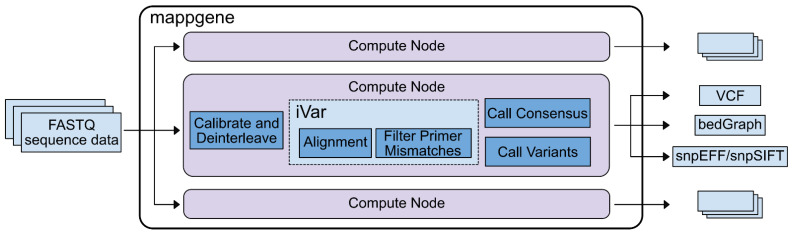
**Mappgene pipeline overview**. The center box shows the mappgene pipeline workflow, and the other 2 computer node boxes above and below the mappgene node signify that mappgene can be run on parallel nodes to process large datasets more rapidly. Note, mappgene uses both iVar and LoFreq to call variants.

Although the ARTIC RT-PCR (1200 bp) protocol was selected to increase consistency of read coverage across regions of the genome that often showed relatively low coverage [13], including much of the S protein ORF, most of the samples had highly variable coverage across the genome with some regions yielding over 1 million x coverage and other regions yielding coverage of less than 100 reads. The high variation and extreme coverage depth can stall bioinformatic analysis pipeline runs of sequence data, even when run on high performance computers. Rapid and thorough analysis of this dataset necessitated the assembly of a specialized bioinformatic pipeline that took into consideration variation in the amount of sequencing coverage across the genome output by this combination of PCR protocol and Illumina platform. This new bioinformatics pipeline, Mappgene, is designed to process a large number of deeply sequenced samples and detect low-frequency variants with high confidence when supported with high read coverage while limiting iSNV calls to higher frequency in regions of lower read coverage (Figure 1). Building on previous work developing a bioinformatics iSNV pipeline [17], extensions to existing publicly available software were made by incorporating LoFreq [18] variant calling into the iVar analysis pipeline [7], a robust bioinformatics pipeline designed for use with the ARTIC primer protocol. Importantly, LoFreq adds the use of additional statistical analysis to enable lower-frequency iSNV (<1%) to be called in regions that have adequate read coverage and quality.

Two samples (#171 and #220) were sequenced as technical duplicates to gauge the consistency of sequencing and bioinformatic results. As expected, this analysis showed all mismatches between duplicates occurred at frequencies lower than 3% [7]. Specifically, the average frequency for the 11 mismatches detected for #171 duplicates was 1.5%, and the #220 duplicates had 0 mismatches. For sample #171, the mutations that were detected in only one of the duplicates occurred in regions of lower coverage, with approximately 80 K average depth of coverage for mutations detected in both samples as compared to 1.5 K average coverage depth for mutations detected in only one duplicate [7]. Sample #171 was selected because it was one of the earliest samples collected in CA (28 February 2020) and had the potential to give unique insight into the earliest mutations that were circulating as the epidemic ignited. However, this sample had the disadvantage of previously being used for a previous study and this increased the number of freeze–thaw cycles which may have impacted RNA quality. Although the C_t_ for this sample was 23, analysis of the mapped reads showed that the coverage across the genome was very uneven with large gaps in coverage. Sample #220 had very good coverage across the genome (>2000× coverage for the vast majority of the genome, with an average of 84,895× coverage), and the C_t_ was 15.

### 3.2. Ranking of iSNVs According to Detection Frequency

iSNV analysis of 90 samples detected 759 unique nonsynonymous mutations (either missense, inframe indels, or premature stop codons) detected in 2 or more samples or in one sample at greater than 3% frequency. For the purposes of this study, an iSNV was defined as a nucleotide that differed from the consensus sequence of the initial strain (NC_045512.2), so that the frequency of detection of this mutation may or may not be <50% in the clinical sample. Thus, in the case of iSNVs detected at >50%, the iSNV would be present as a mutation in the consensus sequence derived from the clinical sample. 

Given the large number of mutations detected, only mutations that resulted in a change in the amino acid sequence (missense mutations) and a subset of in-frame deletions are analyzed in this study. To understand the potential impact of these mutations, the mutations were compared to emergent consensus mutations described in literature and by referencing several highly informative SARS-CoV-2 websites such as outbreak.info, covariants.org, and Nextstrain.org [1,3,19]. 

To determine which of these particular iSNVs are notable in terms of prevalence (# of samples with the iSNV, or “sample count”) and frequency of detection within samples, sample count was multiplied by median frequency of iSNV detection, and mutations were ranked according to these metrics. Additionally, the list of iSNVs and in-frame deletions identified in these samples were queried for the presence of mutations that characterize variants of concern (VOC) and/or variants of interest (VOI).

**Figure 2:**
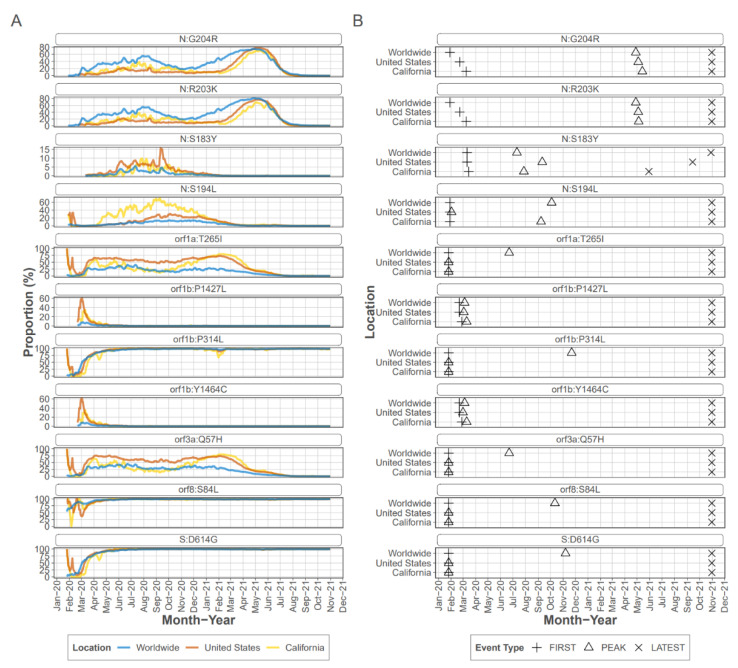
Emergence, prevalence, and duration of highly ranked mutations. (**A**) Prevalence of top-ranked mutations over the course of the pandemic. Each graph represents the 7 day rolling average of the percent positive sequences for each mutation (data obtained from outbreak.info with analysis period through 31 October 2021). (**B**) Emergence, peak and duration of highly ranked mutations. Graphs show for each mutation the date the mutation was detected, the date it reached peak prevalence, and the date last detected. Mutations are considered to be persistent when they are still detected in 1 November 2021, the latest date sequence data available through outbreak.info were analyzed for this study.

Ranking of the mutations that were detected in the CADPH samples according to sample count and frequency revealed that most of the top-ranked mutations were also identified by other studies as prevalent missense mutations detected globally (Table 1) [20]. In particular, the 11 top-ranked mutations were detected at an average frequency ranging from 39 to 98% and were also detected in consensus sequences from early in the pandemic. In some cases, top-ranked mutations were founding mutations for clades that expanded as the pandemic progressed. For example, ORF1b P314L (ORF1ab 4715) was a founding mutation of PANGOLIN B.1 lineage [21] and was detected in 70 samples at an average frequency of 95.2%. Similarly S protein mutation D614G, an early mutation which rapidly became dominant early in the pandemic (Figure 2), was detected in most samples as were the other mutations known to accompany D614G, a C-to-T mutation in the 5′ UTR 241 [20] and ORF1b P314L (nsp12 P323L). 

Four of the top eleven ranked mutations occurred in the N protein, with all occurring within the linker region of the protein which encompasses residues 174 to 249 [22]. Mutations R203K and G204R are present in consensus sequences from early in the pandemic, and were detected as iSNV in 43 CADPH samples each, at frequencies ranging from 1% to >99% from early March to mid-July. Mutations R203K and G204R persisted in the consensus sequences from many lineages and are consistently present in VOC/VOI [23]; however, mutations S183Y and S194L peaked and then decreased in prevalence. Mutation S183Y was seldom detected in consensus sequences collected in CA or the US early in the pandemic and briefly peaked at approximately 10% in late summer of 2020 (outbreak.info). The S183Y iSNV was detected in 20 CADPH samples at 6% to almost 100% from mid June through the latest collection date in July 2020 indicating that this iSNV was frequently present during this early stage of the pandemic. Mutation S194L was infrequently detected in early consensus sequences from CA but peaked at approximately 70% by fall 2020 before declining dramatically by spring of 2021 (outbreak.info). This mutation was detected as an iSNV in 41 CADPH samples from early March to mid-July at frequencies ranging from 1 to nearly 100% suggesting that this mutation is frequently generated and is not likely to reduce genotype fitness. 

**Figure 3:**
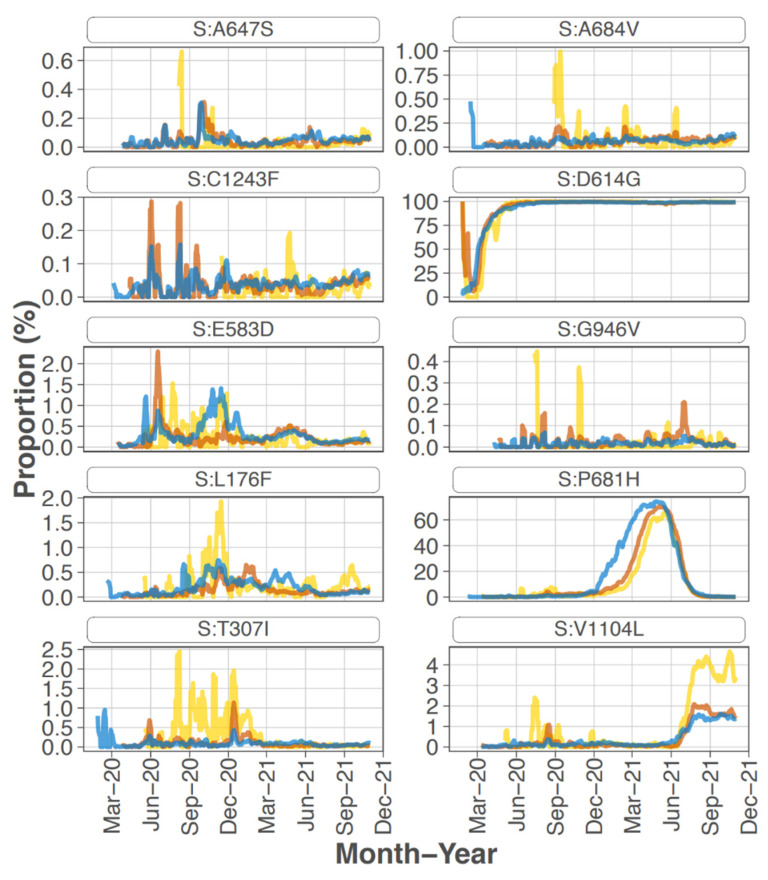
Prevalence of the highest ranked mutations in the spike gene over the course of the pandemic. Each graph represents the 7 day rolling average of the percent positive sequences for each highest ranked mutation (data obtained from outbreak.info with analysis period through October 2021).

**Figure 4:**
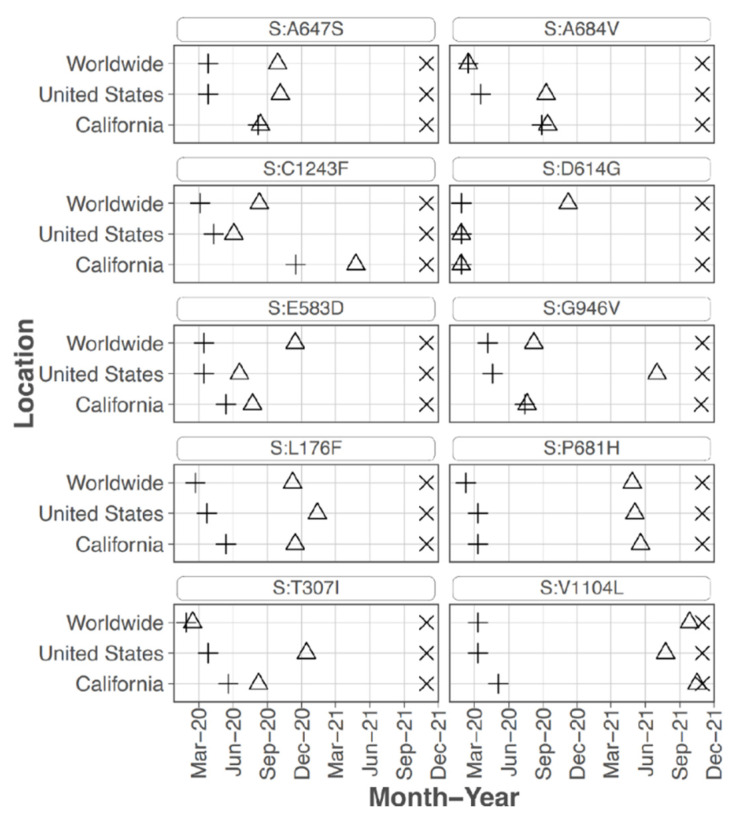
**Emergence, peak and duration of highest ranked spike mutations.** Graphs show for each highest ranked mutation the date the mutation was detected, the date it reached peak prevalence, and the date last detected. Mutations are considered to be persistent when they are still detected in November 2021, the latest date sequence data available through outbreak.info were analyzed for this study.

Focusing on the S protein, iSNVs were ranked according to the sample count and median frequency of the iSNV (Table 2). All the top 10 iSNVs were also present as mutations in consensus sequences on outbreak.info site (Figure 2B, Figure 3 and Figure 4), and persisted into October of 2021 (the date the analysis was performed and the outbreak.info site was queried). Although most of these mutations were subsequently detected only as low-frequency variants in these data from early in the pandemic, three of the mutations, D614G, P681H, and V1104L, emerged in VOC/VOI genotypes months later. 

A subset of the highly ranked mutations such as D614G occur at residues known to impact S protein phenotype. In particular, mutations were detected in 3 of the amino acids in or directly adjacent to the S protein furin cleavage site at the following residues -mutation (number of samples, average frequency): P681H (11, 29.5%), R683Q (1, 8.0%), and A684V (2, 99.5%). Two of these mutations, P681H and A684V were highly ranked. 

### 3.3. Mutations Associated with Voc and/or Voi

Some of the mutations detected in these samples were already well established as dominant within the January to February 2020 time frame, thus detection of these mutations as iSNV could not be considered predictive of later emergence even though these mutations are present in most VOC/VOI genotypes. Notably, none of the mutations that later defined the VOI or VOC genotypes such as N501Y or E484K were detected at this early stage of the epidemic. However, some trends in mutation emergence were noted particularly in the S protein, with a subset of mutations being detected as high frequency iSNV in multiple samples prior to widespread emergence. 

The S protein residue D614G mutation, which rapidly became dominant early in the pandemic, was present in 68 out of 90 samples at an average frequency of 97.8%. Samples without this iSNV detected were all collected in the first months of the pandemic, February and March 2020, with one exception (#231 collected 22 June 2020). All samples collected May to July 2020 except for sample #231, had 614G at >80% frequency. The 2 samples that had 614G detected at <80% occurred in Feb and March at the following percentages and collection dates (in parentheses): 5.3 (11 March 2020) and 52.1 (15 March 2020). Note, no samples from March 16 to May 13 were processed. Thus, the presence and frequency of this iSNV reflected the emergence and establishment of 614G.

S protein P681H was detected in 11 samples at an average frequency of 29.5% (median of 18.1%), and in two additional samples detected by the LoFreq variant caller at less than 1% frequency. All samples with this iSNV were collected within the last month of the study period (29 June 2020 to 20 July 2020). The 681H mutation was first detected in CA on 2020/03/11 but was at low prevalence until late 2020 (outbreak.info 30 September 2021) (Figure 3). The P681H mutation is present in multiple clades and VOC/VOI genotypes Alpha, Gamma, Theta, and Omicron.

S protein mutation H655Y was detected in 31.76% of reads from one sample collected in mid-March 2020 (#210 date 13 March 2020) which is a few months before this mutation was detected as consensus sequence in CA in 4 June 2020 (outbreak.info). It is found in the Gamma VOC genotype and most recently in the Omicron VOC.

Sixteen samples had S protein mutation V1104L detected at an average frequency of 17.07%. The sample collection dates ranged from 15 March 2020 to 20 July 2020; however, 15 of the 16 samples were collected later in this study, between 22 June 2020 and 20 July 2020. The V1104L mutation that recently emerged in many sequences in the Delta 21J clade (AY.36 lineage) and is notable in its relative increase in prevalence in summer of 2021 [1,3]. This residue falls within a T cell epitope [24] and may increase protein stability [25].

E protein mutation P71L, a mutation present in the Beta VOC genotype, was detected in 7 CADPH samples collected between mid-May to mid-June 2020 at an average frequency of 42.9%. Data from outbreak.info show that it was first detected in consensus sequences from CA on 28 March 2020 and continued to be detected at a low frequency (<1%) until there was a brief and minor spike in spring 2021. 

### 3.4. Detection of Rare Mutations Using the LoFreq Variant Caller

A deletion in the S protein between residues 141-145 is commonly found in some variant genotypes. For example, the Alpha, Eta, and Omicron variants have deletions of two or more amino acids at this site. Analysis of LoFreq data show that there is a deletion of residues 141–144 in 11 samples with an average frequency of 0.11%, and a deletion of residue 145 in 25 samples with an average frequency of 0.12%. 

### 3.5. Analysis of Impact of Mutations on Antiviral Drugs

iSNV analysis can also be useful for early detection of mutations that may confer resistance to antiviral drugs, thus the SARS-CoV-2 mutations of interest detected in CADPH data were cross-checked against binding pockets of interest reported [26]. Overlap between the mutations of interest and pockets of interest was observed at the following sites: L214, L373, and G954 in nsp3; Q189 in nsp5; and Y28, E154, L176, R237, P463, E471, V722, D796, L828, A956, L981, T1009, E1017, Q1071, F1103, and D1163 in the S protein. No overlapping sites were observed in nsp12. A comprehensive search of compounds targeting SARS-CoV-2 and other viruses was performed to assess their predicted interaction with the overlapping sites, which could lead to antiviral resistance. For each pocket of interest containing an overlapping site, PDBspheres modeling of protein–ligand interactions [27] was used to identify ligands that are predicted to bind to the pocket. Each of these ligands was compared against the compounds in DrugBank [28] based on Tanimoto similarity, with scores between 0.8 and 1.0 considered a match. Encouragingly, current SARS-CoV-2 compounds were not found to interact with rare variant sites of concern in nsp3 and Spike. Even though antivirals developed against SARS-CoV-2 (GC-373, GC-376, and PF-07304814) were observed to interact with a mutation of interest in nsp5, the mutation (Q189) is so rare that this interaction is unlikely to present a realistic concern. At the same time, a small collection of compounds targeting other viruses (telaprevir, rimantadine, amantadine, and umifenovir) were found to interact with Spike and nsp5 variants, indicating candidate therapeutic small molecules structures to avoid. Altogether, we conclude that rare variant samples do not point to any immediate concerns.

## 4. Discussion

RNA viruses evolve as a genetically diverse intra-host population (“mutant spectrum” or “quasispecies”) and the diversity at the population level may determine the outcome of the infection. Although studies indicate that iSNVs generally occur at frequencies low enough to preclude inter-host transmission [29,30], deep sequencing of viral quasispecies has been used to identify mutations that are present in clinical samples and serve as a source for the emergence of variant genotypes [31]. In particular, iSNVs that are consistently generated over time may become transmitted due to changes in host environment such as persistent infection of an immunocompromised host or cross species transmission [32,33]. Thus, deep sequencing data may reveal which variants are poised to emerge, and influence outbreak trajectory.

Deep sequencing of clinical and wastewater samples can provide insight into the emergence and persistence of mutations that may not be apparent in analysis of consensus sequence data; however, the methods used for detection of low-frequency mutations vary in sensitivity and specificity [34] In particular, the accuracy and sensitivity of detection of low-frequency variants are impacted by sequencing technology, depth of coverage, viral genome copy number, and variant calling algorithms [35] To address these challenges, we limited analysis to samples with at least 1000 viral genomes and employed two different variant callers, including LoFreq which uses statistically rigorous filter for variants occurring at less than 3% due to sequencing error [18].

The CADPH samples analyzed in this study were collected during the first several months of the pandemic, thus the iSNVs were generated largely in response to interaction of the virus (mutant spectrum) with immunologically naïve hosts. This limits the selection pressure of the adaptive immune response on the S protein, although previous exposure to endemic CoVs such as OC43 could potentially influence immune recognition of conserved regions of SARS-CoV-2. In this host environment, the measure of “success” in terms of proliferation and transmission of variant genotypes with particular mutations may be determined according to factors such as viral interaction with the host innate immune response and viral replication rate. 

This study detected many of the mutations that emerged and became established within the first few months; therefore, detection of these mutations as iSNV could not be considered predictive of later emergence. The speed of this emergence and establishment is most apparent in the rapid conversion of the original “wild-type” sequence of strain Wuhan-Hu-1 (NC_045512.2) to the sequence characterized by the S protein D614G mutation that rapidly arose to dominate the first year of the pandemic. The D614G mutation was detected in 76% samples from this study at an average frequency of 98%. Most samples without this iSNV detected were collected in the first months of the pandemic, February and March 2020, whereas all samples but one were collected late in this study (May to July 2020) had the mutation at >80% frequency. Thus, the presence and frequency of this iSNV reflected the emergence and establishment of 614G.

Due to the large number of mutations detected, iSNVs were ranked for further analysis by multiplying the number of samples with the mutation (sample count) by the median frequency the iSNV was detected within a sample (Table 1). Not surprisingly, many of the mutations associated with early expansion of the virus globally were also detected as iSNV in these CA samples from early in the pandemic. For example, ORF1b P314L and S protein D614G were ranked 1 and 2. These rankings agree well with previous analysis of mutational dynamics in the United States. A study by Wang et al. [36] grouped most of these high-ranking mutations as co-occurring, with ORF1ab T265I (nsp2 T85I), ORF1b P314L, S protein D614G, ORF3a Q57H occurring together along with ORF8 S24L, which was not detected in our study. A subset of strains with these mutations also included co-occurring with N protein mutations R203K and G204R. 

Although many of the highly ranked mutations persisted as the pandemic progressed, some highly ranked mutations peaked relatively early in the pandemic but did not persist, such as ORF1b Y1464C and P1427L, ORF8a L84S, and N protein S183Y and S194L. In particular, ORF1b, Y1464C and P1427L, and ORF8 L84S emerged early as part of clade 19B (Nextstrain.org designation) which includes the reference strain, but quickly disappeared as this clade was replaced by other clades. Interestingly, a study by Zhang et al. [37] predicted a deleterious effect of this cluster of mutations on protein function. 

The iSNVs detected were queried for the presence of mutations that characterize the emergence of various VOC and VOI. Variant genotypes such as Alpha and Beta were detected approximately 4 months after the latest samples were collected for this study. Although the collection timeframe may not have been optimal for detection of variant genotype emergence, several mutations that were later associated with variant genotypes were detected as iSNV in some of the samples. Most of these mutations are in the S or N protein; however, E protein mutation P71L was detected in seven samples at average frequency of 43% and is present in the Beta VOC genotype, and the ORF3a Q57H mutation emerged later as part of the genotype of Beta VOC, Epsilon former VOC, and Mu VOI. N protein mutations R203K and G204R were detected as co-occurring mutations and are present in most VOC/VOI. The most prominent N protein mutations were located in the linker region of the protein, and this region has been shown to be integral for RNA binding and viral replication [23]. 

Several iSNV detected in the S protein are associated with VOC and VOI and may impact viral phenotype and most of these were highly ranked (Table 2). Mutations P681H which was detected at high frequencies in 11 samples (average frequency of 29.5%) and is present in Alpha, Gamma, Theta, and Omicron variants. It is located near the furin cleavage site, and may be important for antibody recognition [38]. The addition of a positive charged residue may increase cleavage at the S1/S2 site and affect virus tropism [9,39,40,41]. Detection of this mutation in 12.2% of the samples (11/90) distinguished this iSNV and served as an indicator that this mutation was consistently generated and fit enough to consistently reach relatively high levels within the intra-host population. 

S protein H655Y, which was ranked 14 of spike mutations, is present in Gamma and Omicron variants and was detected at high frequency from one sample from March 2020. This mutation was shown to escape monoclonal antibodies [28], enhance viral replication, S protein cleavage, and transmission [29,30]. Spike V1104L was detected mostly in later samples at an average frequency of 17.1% and later emerged in many Delta variant sequences. Interestingly, S protein substitutions V1104L and H655Y have been associated with cross-species transmission [32,38].

Three residues in or directly adjacent to the S protein furin cleavage site had high-frequency mutations (P681H, R683Q, A684V); however, none of these mutations disrupt the RXXR cleavage motif required for furin protease activity [42]. Additionally, residues S680 through R683 directly contact the T-cell receptor [22] thus mutations at these sites may impact immune response. 

Bioinformatic analysis of nucleotide positions with very high coverage using the LoFreq variant caller enabled detection of very low frequency iSNV that later emerged as part of variant genotypes. In particular, deletions in residues 141–144 and 145 of the N terminal domain of the S protein, was detected at frequencies below 1% in 12.5% and 28.4% of the samples, respectively. The prevalence of these deletions, even at less than 1% frequency indicates the propensity of this region to generate deletions early in the pandemic. Importantly, mutations in this region of the S protein have been shown to impact antibody binding [10,43].

Deep sequence analysis of these early pandemic samples did not detect many of the signature mutations that later emerged months in VOC and VOI such as S protein mutations N501Y, L452R, P681R or E484K. A limitation of this study is that only samples from the first several months of the pandemic were analyzed and these samples were collected from a limited geographical region. As the pandemic further progressed and spread to a global population, opportunities for emergence of even rarely generated mutations increased as did the selection pressure on the viral genome, in particular the spike gene, as a greater proportion of the global population generated an immune response to the virus. For example, infections of immunocompromised patients may be more likely to generate heavily mutated versions of the viral genome [44,45,46]. Future studies will include analysis of deep sequencing data from samples collected later in the progression of the pandemic to understand if signature mutations for VOC/VOI can be detected as rare variants prior to detection in consensus sequence data. 

## 5. Conclusions

Deep sequencing analysis of samples collected in the first several months of the pandemic detected mutations that later emerged in consensus sequences as part of variant genotypes. The majority of the iSNVs detected in these early pandemic samples were also present in consensus data but generally in a relatively low number of sequences. However, most of the mutations were persistently detected over time indicating that they were generated consistently and are sufficiently fit to occasionally become dominant and transmit between susceptible hosts. This indicates that deep sequencing can define reservoirs of a subset of mutations present at various stages of a pandemic [47]. While the predictive capacity of this information may be constrained by temporal and geographical limitations associated with the samples analyzed, deep sequencing data may be useful for understanding which residues and particular amino acid substitutions circulate continuously and may be available to emerge in response to environmental changes such as host immune response. Additionally, because intra-host deep sequencing datasets reveal which genome regions are not observed to mutate, even at the subconsensus level, these data may be predictive of regions that are conserved and unlikely to mutate in the future.

## Figures and Tables

**Table 1:** Top 10 mutations as ranked according to sample count and frequency. Mutations were ranked by multiplying the sample count by the median detection frequency. N protein mutations R203K and G204R are considered co-occurring. NT POS: nucleotide position; SAMPLE COUNT: number of samples with the mutation; AVG FREQ: average frequency; MED FREQ: median frequency; REF NT: nucleotide in the reference sequence; ALT NT: nucleotide in the sample sequence; AA MUT: residue number in the gene with the amino acid mutation and the amino acid change.

NT POS	Sample Count	Rank	AVG FREQ	MEDIAN FREQ	REF NT	ALT NT	AA MUT	Gene
14408	70	69.98	0.952	1.000	C	T	P314L	orf1b
23403	68	68.00	0.978	1.000	A	G	D614G	S
25563	40	31.04	0.616	0.776	G	T	Q57H	orf3a
1059	42	25.99	0.547	0.619	C	T	T265I	orf1a
17858	18	17.95	0.831	0.997	A	G	Y1464C	orf1b
28144	17	16.98	0.895	0.999	T	C	L84S	orf8
17747	17	16.88	0.887	0.993	C	T	P1427L	orf1b
28821	20	16.14	0.651	0.807	C	A	S183Y	N
28854	41	12.34	0.413	0.301	C	T	S194L	N
28883	43	10.35	0.394	0.241	G	C	G204R	N
28881	43	10.23	0.392	0.238	G	A	N203R	N

**Table 2:** Highest and lowest-ranked S protein mutations. The 71 mutations detected in the S protein were ranked by multiplying the sample count by the median detection frequency. NT POS: nucleotide position; SAMPLE COUNT: number of samples with the mutation; AVG FREQ: average frequency; MED FREQ: median frequency; REF NT: nucleotide in the reference sequence; ALT NT: nucleotide in the sample; sequence; AA MUT: residue number in the gene and the amino acid mutation. Gene Regions are noted within the S1 subunit: NTD: N-terminal domain; FCS: furin cleavage site (within or directly adjacent); S1: Spike subunit 1; S2: Spike subunit 2.

NT POS	Sample Count	Rank	AVG FREQ	MEDIAN FREQ	REF NT	ALT NT	AA MUT	Gene Region	Rank #
23403	68	68.0	0.978	1.000	A	G	D614G	S1	1
22088	6	2.5	0.478	0.410	C	T	L176F	NTD	2
23613	2	2.0	0.995	0.995	C	T	A684V	FCS	3
23604	11	2.0	0.295	0.181	C	A	P681H	FCS	4
24872	16	1.4	0.171	0.090	G	C	V1104L	S2	5
22482	10	1.3	0.253	0.131	C	T	T307I	S1	6
23311	2	1.0	0.500	0.500	G	T	E583D	S1	7
23501	1	1.0	1.000	1.000	G	T	A647S	S1	8
24399	1	1.0	0.998	0.998	G	T	G946V	S2	9
25290	2	0.8	0.404	0.404	G	T	C1243F	S2	10

## Data Availability

Accession numbers will be provided prior to publication.

## Supplementary Materials

The following supporting information can be downloaded at: https://www.mdpi.com/article/10.3390/v14122775/s1, Figure S1. SARS-CoV-2 genome structure; Figure S2. Mappgene pipeline components and data processing.
