# Supplementary material for: Multiple Mutations Associated with Emergent Variants Can Be Detected as Low-Frequency Mutations in Early SARS-CoV-2 Pandemic Clinical Samples
Source: Viruses. 2022 Dec 13;14(12):2775. doi: 10.3390/v14122775 (PMC9788161; doi:10.3390/v14122775)
Supplement: Supplementary file 1 [file viruses-14-02775-s001.zip › viruses-1875302-supplementary.pdf]

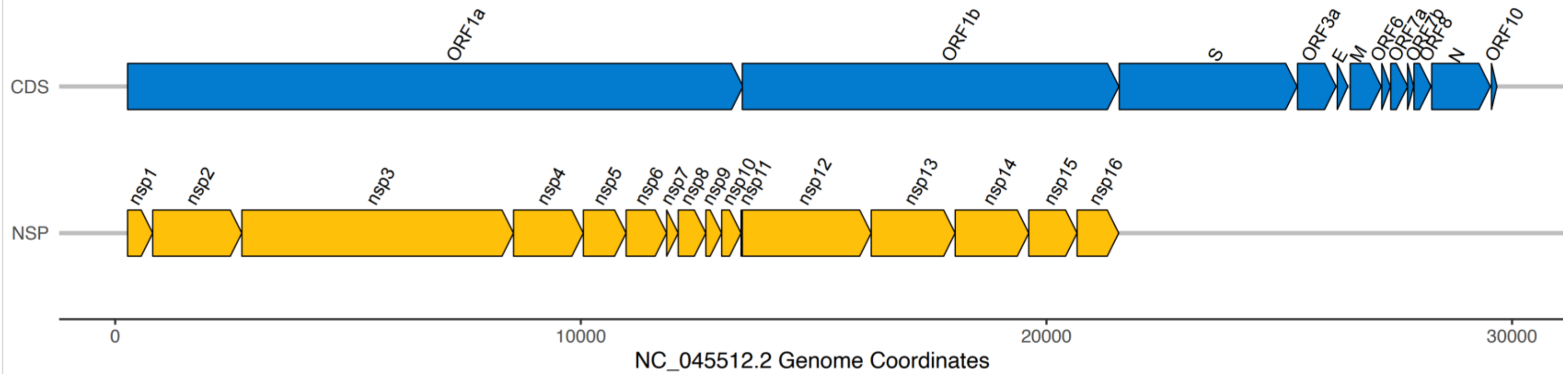

Figure S1. SARS-CoV-2 genome structure

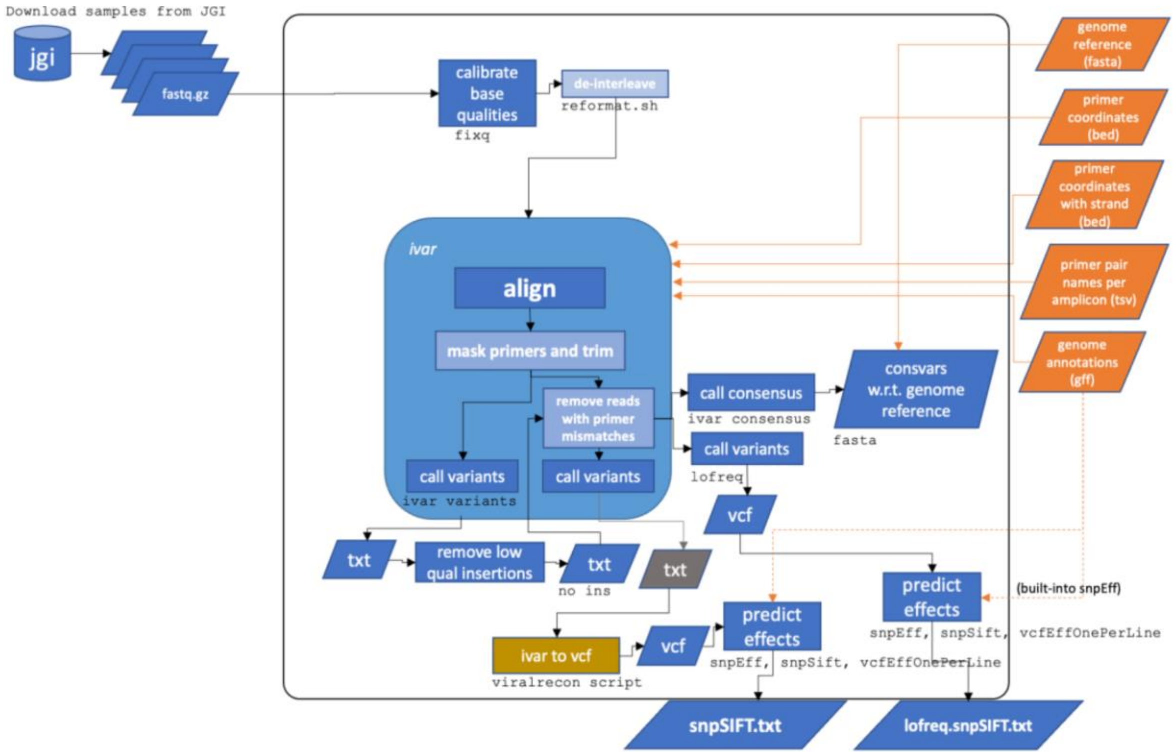

Figure S2. Mappgene pipeline components and data processing (https://github.com/LLNL/mappgene)
